# Association between reduced bronchodilatory effect of deep inspiration and loss of alveolar attachments

**DOI:** 10.1186/1465-9921-6-55

**Published:** 2005-06-08

**Authors:** Nicola Scichilone, Andreina Bruno, Roberto Marchese, Antonio Maurizio Vignola, Alkis Togias, Vincenzo Bellia

**Affiliations:** 1 Istituto di Medicina Generale e Pneumologia, Cattedra di Malattie dell'Apparato Respiratorio, Università di Palermo, via Trabucco 180, 90146 Palermo, Italy; 2 Istituto di Biomedicina ed Immunologia Molecolare, Consiglio Nazionale delle Ricerche, Via Ugo La Malfa 153, 90146 Palermo, Italy; 3 Division of Allergy and Clinical Immunology, and Division of Respiratory and Critical Care Medicine, Department of Medicine, Johns Hopkins University, School of Medicine, 5501 Hopkins Bayview Circle 21224, Baltimore, Maryland, USA

## Abstract

**Background:**

We have previously shown that the bronchodilatory effect of deep inspiration is attenuated in individuals with COPD. This study was designed to investigate whether the impairment in this effect is associated with loss of alveolar attachments.

**Methods:**

We measured deep inspiration (DI)-induced bronchodilation in 15 individuals with and without COPD (67 ± 2.2 yrs of age, mean ± SEM) undergoing lobar resection for peripheral pulmonary nodule. Prior to surgery, we measured TLCO and determined the bronchodilatory effect of deep inspiration after constricting the airways with methacholine. The number of destroyed alveolar attachments, as well as airway wall area and airway smooth muscle area, were determined in tumor-free, peripheral lung tissue.

**Results:**

The bronchodilatory effect of deep inspiration correlated inversely with the % destroyed attachments (r = -0.51, p = 0.05) and directly with the airway smooth muscle area (r = 0.59, p = 0.03), but not with the total wall area (r = 0.39, p = 0.15).

**Conclusion:**

We postulate that attenuation of airway stretch due to loss of alveolar attachments contributes to the loss of the bronchodilatory effect of lung inflation in COPD.

## Background

We have recently demonstrated that the ability of deep inspirations to dilate constricted airways is impaired in subjects with COPD [1]. We have suggested that the lack of deep inspiration-induced bronchodilation may be one of the major factors that contribute to persistent airway narrowing in chronic obstructive pulmonary diseases. However, the mechanism accounting for the reduction in the bronchodilatory effect of deep inspiration in COPD has not yet been elucidated.

When a deep inspiration takes place, radial traction is applied to the outer airway walls by virtue of the forces of interdependence between the airways and the surrounding parenchyma [2], which are sustained by the connective tissue of the lungs. As a consequence, lung inflation produces transient airway distension. If the airways are constricted, the stretch imposed by airway distension may produce bronchodilation [3,4].

The loss of the bronchodilatory effect of lung inflation in COPD may result from factors that unlink the parenchyma from the airways. COPD is accompanied by destructive changes of alveolar walls and consequent reduction in the number of alveolar attachments on the airways [5-7]. We postulated that, because of the alveolar wall destruction, mechanical decoupling between airways and parenchyma results in diminished airway wall stretch, thus impairing the postulated primary step in the mechanism of bronchodilation by deep inspiration. The current study showed that the reduction in alveolar attachments on the airways correlates with the reduction in the bronchodilatory ability of deep inspiration.

## Methods

In order to obtain lung tissue for morphometric and correlative analyses, we enrolled subjects undergoing lobar resection for peripheral pulmonary nodule, with the assumption that a significant proportion would have been smokers and would presumably show evidence of reduced integrity of alveolar attachments [6]. Subjects were recruited from the Unit of Thoracic Surgery, "V. Cervello" Palermo Hospital, Italy. After giving their informed consent, subjects were referred to the Istituto di Medicina Generale e Pneumologia, Palermo University, for potential participation in this protocol. Exclusion criteria for participation in the study were a large involvement of the lung and/or mediastinal organs by the tumor, a history of myocardial infarction, congestive heart failure, arrhythmia, and any unstable clinical condition, such as bronchial exacerbations. The diagnosis of COPD was made according to the diagnostic criteria of the GOLD (Global Initiative for Chronic Obstructive Lung Disease) guidelines [8]. Several subjects with COPD were using bronchodilators and three subjects were using inhaled corticosteroids. The study was approved by the local Ethic Committee.

### Study design

#### Clinical and functional assessment

Prior to surgical lung resection, each subject underwent a clinical evaluation and a complete respiratory functional assessment that included spirometry, and determinations of lung volume and of the single-breath CO diffusing capacity.

The clinical evaluation included a questionnaire that derives from the IUALTD (International Union Against Lung and Tuberculosis Disease) bronchial symptom questionnaire [9] and a physical examination. Total lung capacity (TLC) was determined by bodyplethysmography (Sensor Medics Corporation V6200 AutoBox, Yorba Linda, CA). TLC was expressed as percent predicted based on the prediction equation of Goldman and Becklake [10]. Single-breath diffusing capacity for CO was determined using a fully-computerized water-sealed Stead-Wells spirometer (Baires System; Biomedin, Padua, Italy) and the transfer factor of the lung for CO (TLCO) was measured. At least two determinations of TLCO that were within 5% of each other were obtained, and the highest value was retained for analysis.

**Figure 1:**
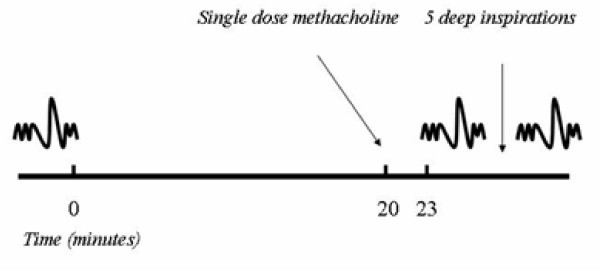
Schematic of the single dose methacholine bronchoprovocation protocol designed to induce at least a 15% reduction in inspiratory vital capacity (IVC), in the absence of deep breaths, and to calculate the bronchodilatory effect of deep inspirations. The combination spirometric maneuvers (partial forced expiration followed by full forced expiration) used to determine IVC are also depicted.

In a series of subsequent visits, the bronchodilatory effect of deep inspirations was determined, as previously described [1,11]. Inhaled short-acting β-agonists and/or anticholinergic agents were withheld for at least 8 h and long-acting β-agonists for at least 24 h before each visit that involved either lung function or methacholine bronchoprovocation. A series of single dose methacholine bronchoprovocations (a single dose per challenge) were performed, in the absence of deep inspirations, using stepwise increasing doses of methacholine. All subjects started with 0.025 mg/ml and increased the dose by a factor of 2 at each visit until an at least 15% reduction in inspiratory vital capacity (IVC) from baseline, under deep breath prohibition (Figure 1). Methacholine was delivered through an ampul-dosimeter (Mefar Elettromedicali; Bovezzo, Italy), which was activated by an inspiratory effort for 0.5 seconds at a time.

To measure IVC, the subject forcefully expired from end-tidal volume to residual volume (partial expiratory maneuver) and immediately inhaled to total lung capacity (IVC = TLC – RV). The maneuver continued with a forced expiration to RV. This forced expiration allowed us to also calculate FEV_1 _and FVC. At baseline, 3 acceptable combined partial/maximal forced expiratory maneuvers were performed, and the best was retained for analysis. Subjects were then instructed to abstain from taking deep breaths for a period of 20 minutes. Thereafter, the single dose of methacholine was administered as five tidal breaths followed, 3 minutes later, by a single partial/maximal combined spirometric measurement, as described above. If the targeted reduction in IVC (at least 15%) was not attained, another single dose challenge was performed. This was done during the same session (at one hour) if the reduction in IVC was within 5% of baseline, or postponed to the next day. Single dose provocations with increasing doses were conducted in this manner until the expected level of reduction in IVC was reached. The provocation in which the targeted reduction in IVC from baseline was attained was extended with 4 deep inspirations taken immediately after the post-methacholine IVC spirometry. Following these deep inspirations, another IVC maneuver was performed. The difference between the IVC obtained after the 4 deep inspirations and the post-methacholine IVC that preceded the 4 deep inspirations was used to calculate the bronchodilatory effect of deep inspirations. This was expressed as percent change from the post-methacholine IVC (% bronchodilation).

We have used IVC in studying the effects of deep inspiration because, assuming that TLC does not change [12], the primary determinant of a change in IVC is the change in residual volume. Although IVC is sensitive to the effects of deep inspirations, its actual measurement, in contrast to that of FVC or FEV_1_, is not influenced by a lung inflation maneuver because RV is reached through a partial forced expiration. FEV_1 _and FVC data were utilized in secondary analyses.

#### Morphometric assessment

For tissue morphometry, we applied the methodology previously described by Saetta and colleagues [6]. Two to seven randomly selected tissue blocks were taken from the subpleural parenchyma of the resected lobe that was tumor-free. Specimens were fixed in 10% neutral buffered formalin (pH 7.2) for at least 24 h and embedded in paraffin wax. Four-μm sections were attached to microscope slides pretreated with polylysine solution (Sigma Aldrich). After dewaxing and rehydratation, all slides were stained with haematoxylin and eosin. Tissue samples were coded and evaluated blindly by two independent investigators using a light microscope (Leica, Wetzlar, Germany). The images were analyzed by a computerized system (Quantimet 500 MC software, Leica, Wetzlar, Germany).

All airways with internal diameter ≤ 2 mm were retained for analysis. Non-respiratory bronchioles with incomplete walls at the edges of the sections or with a short/long diameter ratio < 1/3 were excluded. After this selection, each patient had at least four non-respiratory bronchioles suitable for morphometry. In each airway, the external perimeter (P_e_), the internal perimeter along the subepithelial basement membrane (P_bm_), the lumenal diameter (Dl), the external area (Ae), the internal area (Ai), and the muscle area (WAm) were evaluated. The thickness of the nonrespiratory bronchioles (wall area, WAtot) was obtained by the difference between the external area and the internal areas (WA = Ae – Ai). Values of Dl, WAm and WAtot were normalized by dividing for P_bm _[13].

According to the method of Saetta and colleagues [6], alveolar attachments (AAi) were identified as the alveolar septa that extend from the outer wall of the nonrespiratory bronchioles. Those attachments showing rupture or discontinuity were defined as destroyed alveolar attachments (AAd). The number of destroyed alveolar attachments, expressed as a percentage over the total number of alveolar attachments, represented the primary outcome of the study. The data used for analysis were averages of those obtained independently from each the two study pathologists.

#### Data analysis

Linear regression analysis was performed to correlate the bronchodilatory effect of deep inspirations with the morphometric variables obtained from the lung tissue. Secondary analysis using the same approach was employed to assess the relationship between the magnitude of bronchoconstriction that was induced in the absence of deep breaths (in terms of IVC and FEV1) and the morphometric variables. Unpaired t-tests were used to assess differences between groups. In all analyses, two-tailed values of p = 0.05 were considered statistically significant.

## Results

### Descriptive findings

A total of fifteen subjects took part in the study (age: 67 ± 2.2 yrs, mean ± SEM). Seven of them had a diagnosis of COPD, confirmed by our clinical and functional evaluation. No subject received a diagnosis of asthma. Eleven out of the 15 subjects were smokers (69 ± 27 pack-years, mean ± SD). None of the non-smokers received the diagnosis of COPD. Baseline lung function and lung tissue morphometric characteristics for each individual are presented in Table 1.

**Table 1:** Functional and morphometric characteristics of study participants.

	Mean ± SEM	Range
FEV1, % predicted	82 ± 6.2	43–118
FEV1/FVC	0.65 ± 0.03	0.44–0.77
TLC, % predicted	117 ± 14.3	82–164
TLCO, % predicted	74 ± 3.9	55–92
WAtot/P*bm*, μm	80 ± 7.9	52–154
WAm/P*bm*, μm	11 ± 1.6	6–22
AA*d*, %	33 ± 4.0	12–61

The median single methacholine dose required to induce the targeted reduction in IVC, in the absence of deep inspiration, was 25 mg/ml (range: 0.025–75 mg/ml). The % reduction in IVC in the protocol devoid of deep inspiration was 20 ± 1.8% (mean ± SEM). The % bronchodilation by deep inspiration was 4.3 ± 2.1% with a range of -13% to 18%.

### Correlative findings

**Figure 2:**
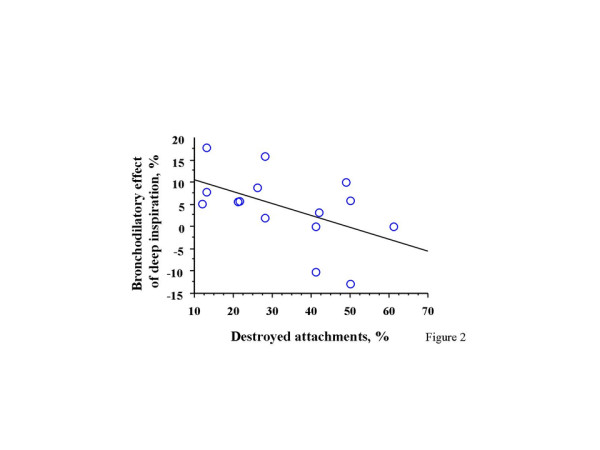
Relationship between the bronchodilatory effect of deep inspiration and the percentage of destroyed alveolar attachments.

**Figure 3:**
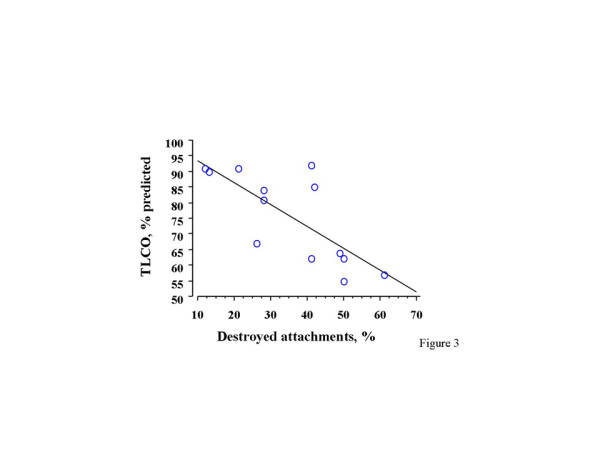
Relationship between TLCO and the percentage of destroyed alveolar attachments.

We found a significant inverse correlation between the bronchodilatory effect of deep inspiration and the percentage of destroyed alveolar attachments (r = -0.51, p = 0.05, Figure 2). In addition, the bronchodilatory effect of deep inspiration correlated directly with the airway smooth muscle area (r = 0.59, p = 0.03). In contrast, no correlation with the magnitude of the total wall area (r = 0.39, p = 0.15) was observed. The multiple regression analysis, in which the bronchodilatory effect of deep inspiration is the dependent variable, and the percent of destroyed alveolar attachments and the airway smooth muscle area serve as independent variables, yielded a p value of 0.02; however, neither the alveolar attachment (p = 0.09) nor the airway smooth muscle (p = 0.06) entered the model. The bronchodilatory effect of deep inspiration did not differ between subjects with COPD and the 4 subjects without COPD, but with a history of smoking (2.6 ± 4.2% vs. 5.2 ± 3.6%, respectively; p = 0.68). Similarly, no differences were found between COPD subjects and the non-COPD smokers with respect to the percentage of destroyed alveolar attachments (40 ± 7.2% vs. 35 ± 3.9%, respectively; p = 0.64). When the entire group was considered, the % destroyed attachments showed a strong inverse correlation with TLCO% predicted (r= -0.75, p = 0.003) (Figure 3).

## Discussion

We have recently documented the lack of deep inspiration-induced bronchodilation in COPD [1]. The results of this study confirm and extend our previous report. Herein, we provide a possible explanation for this phenomenon, by showing that the impairment of the bronchodilatory effect of deep inspiration is associated with reduction in the alveolar attachments to the airway walls.

**Figure 4:**
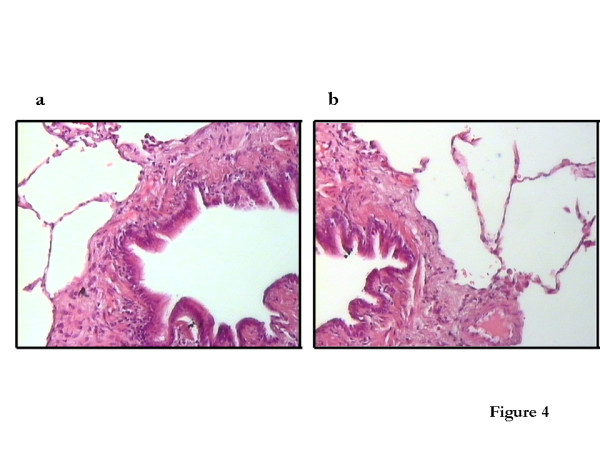
Pathology picture showing the intact (a) and the destroyed (b) alveolar attachments.

A significant correlation is not a proof for a causative relationship, but it is a pre-requisite for it. Moreover, there is good theoretical reason to propose that the reduction in the number of alveolar attachments is the most important factor responsible for the impairment in deep inspiration-induced bronchodilation, in smokers and individuals with COPD. A body of evidence has suggested that the effect of deep inspiration on the airways is a function of the interdependence between the airways and the parenchyma [14,15], provided by the alveolar attachments that act by distending the airways when lung volume increases (Figure 4), and by the relative magnitudes of airway and parenchymal hystereses [16]. According to this theory, equal degrees of hysteresis result in no effect of a deep inspiration on airway caliber. If parenchymal hysteresis prevails, such as in COPD [17], a deep inspiratory maneuver fails to dilate airways, and may even result in bronchoconstriction. Therefore, the impairment in the bronchodilatory effect of deep inspiration in subjects with COPD could be explained by the increased ratio of parenchymal over airway hysteresis.

We reasoned that structural alterations of the lung parenchyma, specifically the destruction of alveolar attachments, would reduce the effectiveness of the distending forces in a manner that a deep inspiration would not be capable of stretching narrowed airways and/or reopen closed airways. However, other explanations need to be considered: first, increased airway smooth muscle mass could render the muscle too stiff to stretch, or generate higher forces that could counteract bronchodilation. However, we found that larger smooth muscle area was related to stronger bronchodilation by deep inspiration. Second, COPD could be associated with enhancement of a bronchoconstriction reflex that is activated by lung inflation, or with the failure to release bronchodilatory agents. Third, under a condition of reduced stretch, the airway smooth muscle could develop peculiar rearrangement of the contractile elements that would induce a state of increased resistance to the effect of deep inspiration. Finally, in a condition of lung hyperinflation, which is often recorded in subjects with emphysema, the amplitude of a deep inspiration could be severely reduced.

Corsico et al. [18] recently reported findings that have similarities to ours. The authors showed that the loss of alveolar attachments is associated with a bronchoconstrictor effect of deep inspiration. In our previous study, which was conducted on subjects with COPD [1], we observed that, in those subjects with the lowest TLCO, deep inspirations led to bronchoconstriction, instead of bronchodilation. We have the same observation in this study (Figure 2), in individuals who are among those with the highest percentage of destroyed alveolar attachments (>40%). In the study of Corsico and coworkers, the percentage of destroyed alveolar attachments is higher than that of the current study (46% vs. 33%). It is plausible that mild parenchymal alterations, such as those observed in smokers [6,19], would attenuate bronchodilation by deep inspirations, whereas more advanced abnormalities of the lung would convert the beneficial effect of deep inspiration into a detrimental one. Whereas the morphometric approach was identical in the two studies, the functional assessment was different, in that, Corsico and colleagues employed the baseline ratio of maximal over partial expiratory flows (M/P), which may be a measure of deep inspiration-induced distensibility, rather than deep inspiration-induced bronchodilation. In other words, our protocol assesses the consequences of the deep inspiratory maneuver after the maneuver is completed, whereas the M/P ratio describes the difference in flow between a partial and a maximal expiration without necessarily predicting what the state of airway will be at the end of the maneuver.

The correlation between the loss of the bronchodilatory ability of deep inspiration and the loss of alveolar attachments becomes even stronger if it is viewed in the context of the fact that the range of deep inspiration-induced bronchodilation that we observed in our subjects was quite narrow (18 to -13%). Overall, bronchodilation was substantially reduced in this group (4.3 ± 2.1%), compared to an average value of around 20% that we would have expected in healthy individuals of the same age, based on our previously published data [11]. It is also important to note that the average bronchodilation by deep inspiration we report in this group of subjects is the same as in a group of individuals, all diagnosed with COPD, that we have reported earlier [1]. Although the number of subjects is too small for meaningful conclusions to be drawn, it is interesting that the deep inspiration effect appeared to be reduced even in smokers without the diagnosis of COPD. In our previous study on subjects with COPD, the bronchodilatory effect of deep inspiration correlated with TLCO, but not with spirometric outcomes such as FEV_1 _or FEV_1_/FVC [1]. The significant inverse correlation between TLCO and the percentage of destroyed attachments we found in this study offers an explanation for the above-cited relationship.

The lack of correlation between the thickness of airway wall and the attenuation of the bronchodilatory effect of deep inspiration is also in agreement with the report by Corsico and colleagues [18], and indicates that, when parenchymal destruction is present, airway wall factors play a secondary role in determining the magnitude of the beneficial effects of deep inspiration. This may be different in asthma, where parenchymal involvement appears to be minimal [20,21]. The presence of a direct correlation between the bronchodilatory ability of deep inspiration and the airway smooth muscle area is difficult to explain. One possibility is that increased smooth muscle mass leads to more bronchoconstriction and this may, up to a point, increase the bronchodilatory effects of deep inspiration by increasing radial traction [15,22]. Indeed, we have previously shown that the bronchodilatory effect of deep inspiration cannot be measured when the induced bronchoconstriction is relatively small [3].

## Conclusion

The results of our study support the hypothesis that the attenuation of airway stretch due to loss of alveolar attachments represents an important cause for the impairment in the bronchodilatory effect of lung inflation in COPD. Whether the progressive impairment of the beneficial effect by deep inspiration has clinical and prognostic implications in these subjects needs to be addressed in future studies.

## Competing interests

Dr. Togias' participation in this work was supported by NIH grant RO1 HL61277.

The authors declare that they have no competing interest.

## Authors' contributions

NS conceived and designed the study, performed the clinical and functional assessments, analyzed and interpreted the data, drafted the manuscript; AB carried the morphometric assessment and participated to the interpretation of the findings; RM carried the clinical and functional assessments and participated to the interpretation of the data; AMV participated to the design and the coordination of the study and the interpretation of the results; AT conceived and participated to the design of the study, the analysis of the data and the interpretation of the results, and contributed significantly to draft the manuscript; VB helped in the design and the organization of the study, as well as the interpretation of the results.
